# Ovarian Cycle Stages Modulate Alzheimer-Related Cognitive and Brain Network Alterations in Female Mice

**DOI:** 10.1523/ENEURO.0132-17.2018

**Published:** 2018-12-14

**Authors:** Lauren Broestl, Kurtresha Worden, Arturo J. Moreno, Emily J. Davis, Dan Wang, Bayardo Garay, Tanya Singh, Laure Verret, Jorge J. Palop, Dena B. Dubal

**Affiliations:** 1 Department of Neurology and Weill Institute for Neurosciences, University of California, San Francisco, California 94158; 2 Biomedical Sciences Graduate Program, University of California, San Francisco, California 94158; 3 Neurosciences Graduate Program, University of California, San Francisco, California 94158; 4 Gladstone Institute of Neurological Disease, University of California, San Francisco, California 94158

**Keywords:** Alzheimer’s disease, estrogen, hormones, mice, network, sex

## Abstract

Alzheimer’s disease (AD) begins several decades before the onset of clinical symptoms, at a time when women may still undergo reproductive cycling. Whether ovarian functions alter substrates of AD pathogenesis is unknown. Here we show that ovarian cycle stages significantly modulate AD-related alterations in neural network patterns, cognitive impairments, and pathogenic protein production in the hAPP-J20 mouse model of AD. Female hAPP mice spent more time in estrogen-dominant cycle stages and these ovarian stages worsened AD-related network dysfunction and cognitive impairments. In contrast, progesterone-dominant stages and gonadectomy attenuated these AD-related deficits. Further studies revealed a direct role for estradiol in stimulating neural network excitability and susceptibility to seizures in hAPP mice and increasing amyloid beta levels. Understanding dynamic effects of the ovarian cycle on the female nervous system in disease, including AD, is of critical importance and may differ from effects on a healthy brain. The pattern of ovarian cycle effects on disease-related networks, cognition, and pathogenic protein expression may be relevant to young women at risk for AD.

## Visual Abstract

**Figure:**
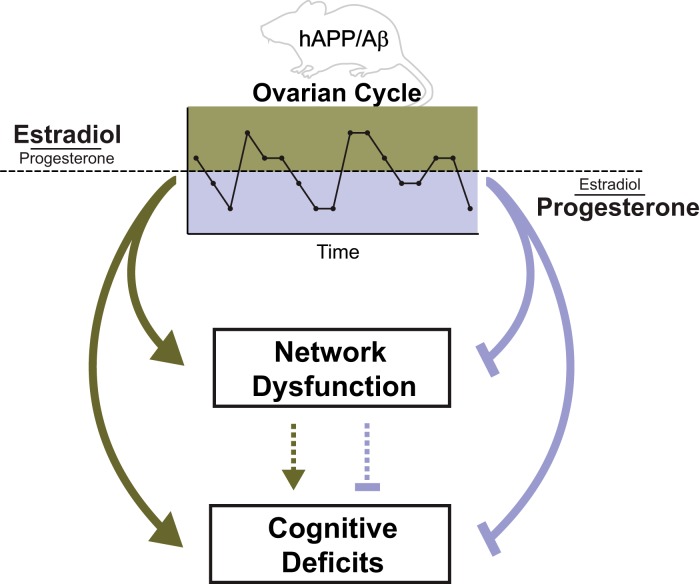

## Significance Statement

Alzheimer’s disease (AD) is insidious and begins several decades before clinical symptoms. For women, this means that pathophysiological changes could occur in the brain during the reproductive life-stages, before the cessation of ovarian function. Whether ovarian functions alter substrates of AD pathogenesis is unknown. We show that ovarian cycles are altered in a mouse model of AD and that ovarian cycle stage contributes to AD-related network and cognitive impairments. Investigating network activity, cognition, seizures, and other manifestations of brain function across the reproductive cycle stages in cycling women could reveal differential patterns in the brain at risk for AD compared with normal aging, and potentially open the door for preventative therapies and early treatment in women at risk for AD.

## Introduction

Alzheimer’s disease (AD) is the most common dementia. Without interventions, >80 million people will suffer from AD by 2050 worldwide (Prince et al., 2013). Altered network activity is one of the earliest alterations in the pathogenesis of AD (Palop and Mucke, 2010, 2016). In mouse models of AD, networks become destabilized, hyperexcitable, and display epileptiform activity (Palop et al., 2007; Minkeviciene et al., 2009; Ittner et al., 2010; Roberson et al., 2011); alterations closely linked with cognitive dysfunction (Verret et al., 2012; Martinez-Losa et al., 2018). In humans, hippocampal hyperactivity (O’Brien et al., 2010; Bakker et al., 2012; Reiman et al., 2012) and altered network connectivity (Pievani et al., 2011; Chhatwal et al., 2013), precede clinical symptoms of AD, predict disease conversion, and correlate with cognitive deficits. Importantly, humans with AD, particularly in familial AD, also have an increased incidence of seizures and epileptiform activity (Palop and Mucke, 2016). Recent studies suggest that the prevalence of these abnormalities among sporadic AD patients may be vastly underestimated (Vossel et al., 2013, 2017). Indeed, hippocampal non-convulsive seizures have been recently demonstrated with intracranial foramen electrodes in AD patients without a history of seizures (Lam et al., 2017).

AD is insidious and begins decades before cognitive and behavioral deficits appear (Bateman et al., 2012; Villemagne et al., 2013). For women, this means that pathophysiologic changes could occur in the brain at risk for AD during the reproductive life-stage, before cessation of ovarian function. Although ovarian functions alter neural networks, effects that fluctuate across the reproductive cycle (Smith and Woolley, 2004; Scharfman and MacLusky, 2006), it is unknown whether ovarian functions alter pathophysiological substrates of cognitive impairment in dementia. Estrogen and progesterone, primary ovarian hormones dominant at different cycle stages, can exert opposing effects on the brain. In wild-type rodents, cycle stages high in estrogen increase excitatory synaptic input (Woolley et al., 1990; Woolley and McEwen, 1992; Scharfman et al., 2003) and enhance learning and memory (Rhodes and Frye, 2004; Walf et al., 2009). In humans, cognition (Maki et al., 2002; Gogos, 2013) and fMRI activation (Fernández et al., 2003) also fluctuate across the ovarian cycle.

Estrogen can promote neural function under normal conditions, but exacerbates dysfunction when network activity is disrupted. In wild-type rodents, estrogen-dominant cycle stages (Terasawa and Timiras, 1968) and exogenous estradiol (Woolley, 2000; Maguire et al., 2005; Ledoux et al., 2009) increase seizure susceptibility, whereas progesterone-dominant cycle stages decrease it (Maguire et al., 2005; Wu et al., 2013). In women with catamenial epilepsy, cycle stages similarly correlate with seizure frequency (Herzog et al., 1997; Reddy, 2013). Because seizures and increased epileptic activity are strongly associated with AD and may even contribute to early pathogenesis of the disease (Palop and Mucke, 2009; Vossel et al., 2017; Edwards and Robertson, 2018), we wondered whether ovarian cycle stages high in estrogen worsen AD-related deficits.

Thus, we examined ovarian cycles in the hAPP-J20 mouse model of AD and determined how they alter network activity, cognitive function, and expression of pathogenic AD proteins. Transgenic mice from the J20 line express a mutant human amyloid precursor protein (hAPP) that causes early-onset AD in humans (Mucke et al., 2000). hAPP mice have elevated levels of hAPP and toxic amyloid-β (Aβ) peptides in the brain and recapitulate key aspects of human AD, including cognitive deficits, behavioral abnormalities, synaptic dysfunction, hyperexcitable neural networks, neuritic amyloid plaques, and increased mortality (Cissé et al., 2011; Dubal et al., 2015).

Here we show that ovarian functions dynamically modulate key AD-related deficits in female mice. Female hAPP mice exhibit normal ovarian cycle duration, but lengthened follicular phases, dominated by estrogen. Cycle stages with a high estrogen/progesterone ratio (High E/P), proestrus and estrus, worsen network and cognitive dysfunction in hAPP mice, whereas cycle stages with a low ratio (Low E/P), metestrus and diestrus, attenuate deficits. Levels of the pathogenic peptide Aβ_1-42_ increase during proestrus, a time of amplified network dysfunction. We also reveal a direct role for a transient estradiol surge in aggravating network hyperexcitability and increasing Aβ_1-42_ levels following seizures in hAPP mice. The pattern of ovarian cycle effects on disease-related networks, cognition, and pathogenic protein expression may be relevant to young women at risk for AD, and may also differ in a normal brain compared to one at risk for AD.

## Materials and Methods

### Animals

Female mice were from the hAPP-J20 line, which expresses a mutant form of the human amyloid precursor protein with the Swedish (K670N/M671L) and Indiana (V717F) familial mutations, under control of the PDGF β-chain promoter (Rockenstein et al., 1995; Mucke et al., 2000). Female mice used in this study were all on a congenic C57BL/6J background, except for a subset of F1 C57Bl/6J and FVBN hybrids used in seizure studies (see Fig. 7*D*; *n* = 4 vehicle and *n* = 3 E_2_). Mice were kept on a 12 h light/dark cycle with *ad libitum* access to food (Picolab Rodent Diet 20, Labdiet) and water. Studies were conducted in age-matched and littermate mice. Each experiment was conducted within a 2.5 month range (or less) in the female reproductive life stage, as indicated in the figure legends. Females used in cognitive tasks, EEG, and biochemical studies were nulliparous. All animal procedures were performed in accordance with the University of California San Francisco animal care committee’s regulations.

### Determination of ovarian cycles

**Figure 1.**
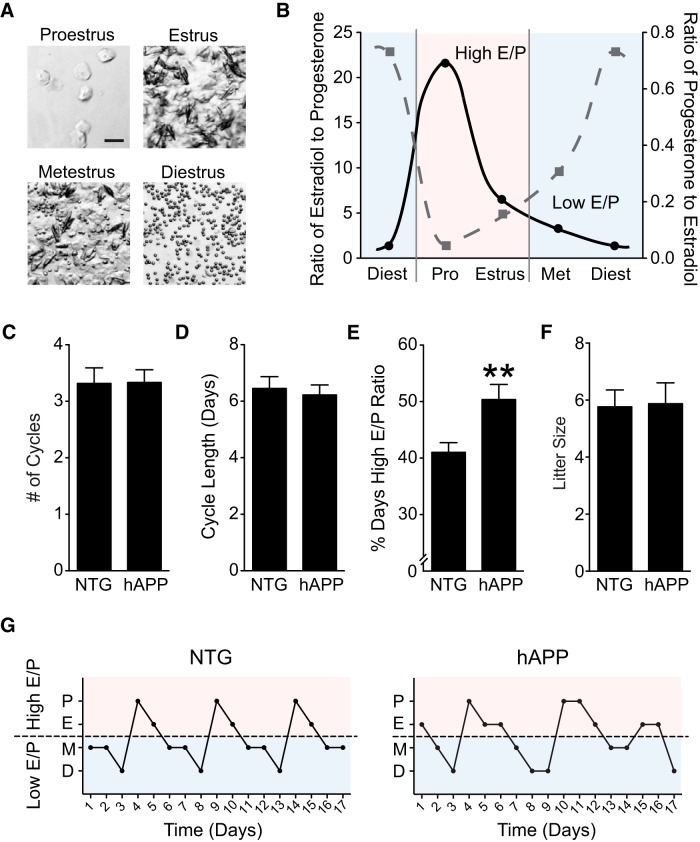
Altered ovarian cycling in female hAPP mice. ***A***, Representative images of vaginal cytology classified as proestrus (nucleated epithelial cells), estrus (cornified squamous epithelial cells), metestrus (mix of cell types), or diestrus (leukocytes). Scale bar, 50 μm. ***B***, Ratio of estradiol (pg/ml) to progesterone (ng/ml; E/P) at each stage of the mouse estrous cycle, adapted from characterized hormone levels (Walmer et al., 1992; Fig. 1-1). ***C***, Quantification of the number of ovarian cycles spanning ∼3 weeks in NTG and hAPP mice. ***D***, Quantification of cycle length in days for NTG and hAPP mice during this same period. ***E***, Percentage time spent in stages of the cycle with a High E/P ratio (proestrus, estrus). **p < 0.01^c^. ***F***, Number of pups per litter born to NTG and hAPP female mice. ***G***, Representative estrous cycles in female NTG and hAPP mice. The dashed line represents a demarcation between High E/P and Low E/P cycle stages (*n* = 12–18 mice per genotype for ***C***–***F***, age 2.5–4 months). Data are mean ± SEM. See also Figure 1-1.

Vaginal lavages were performed daily on female non-transgenic (NTG) and hAPP mice. The vaginal opening was flushed 2–3 times with 50μl normal saline using a transfer pipet and collected on a slide. Slides were manually read at 5x magnification and classified (Fig. 1A) as proestrus (nucleated epithelial cells), estrus (cornified squamous epithelial cells), metestrus (mix of all 3 cell types), or diestrus (leukocytes) by an investigator blinded to the genotype of the mouse. Vaginal cytology was assessed each morning for a period of ∼3 weeks (17–24 d). Number and length of cycles was quantified and a cycle was counted each time proestrus was followed by a successive estrus. Cycle stage for 1–3 sporadic days per mouse was extrapolated based on the preceding and succeeding days of the cycle; analysis of only sampled data did not alter results (data not shown). To quantify the percentage of days spent in a High E/P ratio, each cycle stage was classified based on relative levels of estradiol and progesterone (Walmer et al., 1992). Proestrus and estrus were grouped as High E/P stages, and metestrus and diestrus were grouped as Low E/P stages. The number of days mice spent in proestrus and estrus were counted and divided by total days. All mice, intact and gonadectomized, underwent vaginal lavages to minimize any differences in handling between experimental groups.

10.1523/ENEURO.0132-17.2018.f1-1 Figure 1-1 Estradiol to progesterone ratio across the mouse estrous cycle. ***A***, ***B***, Ratio of estradiol to progesterone at (***A***) each stage of the mouse estrous cycle and (***B***) averaged across High E/P (proestrus and estrus) and Low E/P (metestrus and diestrus) stages. ***C***, Hormone levels used to calculate ratios, adapted from Walmer et al. (1992). Download Figure 1-1, EPS file

### Gonadectomy

Mice were anesthetized with Avertin (400 mg/kg, i.p.) and the surgical area was shaved and prepped with 70% ethanol. Gonadectomy (ovary removal) was performed via bilateral abdominal incisions ∼1.5 cm below the dorsal midline and 1 cm posterior to the end of the rib cage. Following exteriorization and surgical removal of the ovaries, absorbable sutures were used to close the incisions. All surgeries were performed at least 3 weeks before testing.

### Behavior cohorts and brain collection

A separate, independent cohort of mice was used in each behavioral task. In the cohort of mice that underwent passive avoidance testing, brains were collected for studies in Figure 6, following ∼1 week of further ovarian cycle assessment. In the cohort of mice that underwent seizure induction, brains were collected immediately following the experiment.

### Passive avoidance

The apparatus consists of a two-compartment dark/light shuttle box separated by a guillotine door, with a stainless-steel shock grid floor in the dark compartment (Gemini, Avoidance System, San Diego Instruments). During training, mice were placed in the lit chamber and given 15 s to acclimate. After 15 s, the door between the two chambers was raised, and latency to enter the dark chamber was recorded. Immediately after mice entered the dark chamber, the door closed and a foot shock was delivered (0.35 mA, 2 s). Ten seconds following shock, mice were removed and returned to their home cage. Twenty-four hours after training, latency to enter the dark chamber was measured over a maximum time period of 400 s. The apparatus was cleaned with 70% ethanol between each mouse. During analysis, mice that showed a decreased latency during testing compared with training (2 mice from the gonadectomized hAPP group that escaped the apparatus and were not shocked) were excluded.

### Open field

Total activity was measured using an automated Flex-Field/Open Field Photobeam Activity System (San Diego Instruments). The apparatus consisted of a clear plastic chamber (41 × 30 cm) with two 16 × 16 photobeam arrays. Mice were acclimated to the room for 30 min before testing, placed in the chamber, and allowed to explore freely for 10 min while total activity was measured. The apparatus was cleaned with 70% ethanol between each testing session.

### Two trial Y-maze

Mice were acclimated to a dimly lit room for 30 min before testing, and the maze was cleaned with 70% ethanol between each testing session. The Y-maze apparatus was constructed of opaque white plastic and consisted of 3 rectangular arms (length: 49.5 cm, width: 8.0 cm, height: 23.5 cm) 120**°** apart, connected by a triangular center piece; an opaque divider could be slid into the entrance of each of the arms to block them. Distinct visual cues were attached to the ends of two of the arms, while the Start Arm was left blank. During training one of the two visual cue arms was blocked (Novel Arm). Mice were then placed in the Start Arm and allowed to explore the two open arms (Start and Familiar) freely for 5 min. The Novel and Familiar Arms alternated between mice to control for any innate preference the mice might have for one arm. After training, mice were returned to their home cage. Sixteen hours later, mice were returned to the maze with all three arms unblocked. Mice were allowed to explore freely for 3 min, and distance traveled in the Novel and Familiar Arms was measured automatically using the ANY-Maze system (San Diego Instruments).

### EEG recordings

Mice were anesthetized with Avertin (400 mg/kg, i.p.), and Teflon-coated silver wire electrodes (0.125 mm diameter) soldered to a multichannel electrical connector were implanted into the subdural space over the left frontal cortex (1 mm lateral and anterior to the bregma) and the left and right parietal cortices (2 mm lateral and posterior to the bregma). The left frontal cortex electrode served as a reference. EEG activity was recorded for 24 h in freely moving mice in a recording chamber with Harmonie software v5.0b. Spikes were automatically detected using Gotman spike detectors (Harmonie) with an amplitude threshold of 8. Spikes were analyzed from 3:00 P.M. to 5:00 A.M. and graphed before and after the E2 surge, the hours of 15:00–16:00 (3:00–4:00 P.M.) and 03:00–04:00 (3:00–4:00 A.M.).

### Seizures

Mice were gonadectomized ∼3 weeks before treatment and then injected with sesame oil (vehicle; 100 μl, i.p.) or 17β-estradiol benzoate dissolved in sesame oil (5 μg/100 μl, i.p.). This dose of estradiol mimics *in vivo* levels of estradiol during proestrus (Akinci and Johnston, 1997). Twenty-four hours later, pentylenetetrazol (PTZ; 35 mg/kg, i.p.) dissolved in phosphate buffered saline (5 mg/ml) was injected and seizure response was videotaped. An investigator blinded to genotypes and treatments manually scored seizures on an 8-point scale with increasing severity from 1 to 8: Stage 1, pausing; Stage 2, first spasm; Stage 3, tail extensions; Stage 4, forelimb clonus; Stage 5, generalized clonus; Stage 6, bouncing/running seizures; Stage 7, full tonic extension; Stage 8, death. Latency to reach each seizure stage was recorded, with a maximum latency of 1200 s. Seizures were then classified into early and late stages, with late stages representing Stage 4 (beginning of clonic movements) through Stage 8.

### Protein extraction

The hippocampus was microdissected and weighed. Ice-cold lysis-buffer [1× PBS, pH 7.4, 1 mm DTT, 0.5 mm EDTA, 0.5% Triton, 0.1 m phenylmethyl sulfonyl fluoride, protease inhibitor mixture (Roche), and phosphatase inhibitors 2 & 3 (Sigma-Aldrich)] was added to a final concentration of 100 ng tissue/μl, and the tissue was homogenized with a motorized pestle mixing/grinding rod (Kontes), then sonicated. After samples rested on ice 20 min, a 50 μl aliquot was removed for ELISA, and samples were further diluted with an additional 100 μl lysis-buffer, then centrifuged at 9400 × *g* for 10 min at 4°C. The supernatant was collected for Western blot analyses, and protein concentration was measured with the BCA assay.

### Immunoblotting

Twenty micrograms of protein were loaded into each well of a 4–12% Bis-Tris gradient SDS-PAGE gel. Gels were transferred to nitrocellulose membranes and immunoblotted with antibodies against human amyloid precursor protein (8E5, 1:5000; Elan Pharmaceuticals), total tau (EP2456Y, 1:20,000; Millipore), phosphorylated-tau (PHF-1, 1:2000; Peter Davies, Albert Einstein College of Medicine), and actin (A2066, 1:3000; Sigma-Aldrich). Gels were read using the Odyssey CLx Imaging System (LI-COR Biosciences) and quantified using Image Studio Lite software. Actin was used as a loading control and quantified independently to ensure there were no differences between groups (data not shown).

### ELISA

The 50 μl aliquot from the protein extraction step was centrifuged at 18,400 × *g* for 15 min at 4°C. One hundred microliters of 7.5 m guanidine buffer (50 mm Tris, pH 8.0, 7.5 m guanidine-HCl) was added (final guanidine concentratio*n* = 5 m). A motorized pestle mixing/grinding rod (Kontes) was used to break down the pellet, and samples underwent shaking at room temperature for 3 h, and were then stored at −20°C. Levels of Aβ_1-42_ were measured using a solid phase sandwich ELISA (Immuno-Biological Laboratories) according to the manufacturer’s instructions, yielding an average coefficient of variation of 7.82%. Briefly, samples were diluted 10-fold with the supplied Enzyme Immunoassay dilution buffer, then centrifuged at 18,400 × *g* for 20 min at 4°C, and supernatant was collected for loading onto the ELISA plate. A standard curve was prepared by serial dilution of human Aβ_1-42_. Standards and diluted samples (100 μl each) were loaded in duplicate onto a plate precoated with affinity-purified anti-human Aβ (38-42) rabbit IgG antibody, and incubated overnight at 4°C. The plate was washed 7 times with washing buffer, then 100 μl of HRP conjugated anti-human Aβ(N) rabbit IgG affinity-purified antibody was added to each well. Following incubation for 1 h at 4°C the plate was washed nine times, and the reaction was visualized by the addition of 100 μl of chromogenic substrate (TMB) for 30 min at RT in the dark. The reaction was stopped by the addition of 100 μl 1 N H_2_SO_4_, and absorbance at 450 nm was measured using a SpectraMax Plus 384 plate reader (Molecular Devices). Aβ_1-42_ levels were calculated with SoftMax Pro software.

### Quantitative reverse-transcription PCR

RNeasy Mini kits (Qiagen) were used to extract total RNA from hippocampus. Total RNA was reverse transcribed using TaqMan Reverse Transcription Reagents (Applied Biosystems). The expression level of hAPP mRNA was determined using SYBR Green PCR Master Mix and an ABI PRISM 7900HT sequence detection system (Applied Biosystems). Analysis of dissociation curves, standard curve slopes, and reverse-transcription-negative reactions were used to verify the quality of primers and amplification reactions. Expression levels of GAPDH mRNA were used as a control to ensure that levels did not vary between groups (data not shown). Primer sequences: *hAPP* (forward, 5′-GAGGAGGATGACTCGGATGTCT-3′; reverse, 5′-AGCCACTTCTTCCTCCTCTGCTA-3′); *mouse GAPDH* (forward, 5′-GGGAAGCCCATCACCATCTT-3′; reverse, 5′-GCCTTCTCCATGGTGGTGAA-3′).

### Statistics

All experimenters were blinded to genotypes and hormonal status of mice. Statistical analyses were performed with GraphPad Prism v5.0 for *t* tests, two-way ANOVAs, and linear regression, and Stata v13.0 for mixed-model ANOVAs and χ^2^ tests. R (R Foundation for Statistical Computing, 2013) was used to correct for multiple comparisons. *Post hoc* comparisons were corrected for using the Bonferroni–Holm method. Differences among multiple means for unpaired data were assessed by two-way (genotype and ovarian status/treatment), between-subject ANOVAs. A mixed-model ANOVA was used for analyses of spikes (factors: ovarian status and time) and seizure latency (factors: genotype, severity, and treatment). Differences between categorical variables were assessed with Pearson’s χ^2^ test. Error bars represent SEM. Null hypotheses were rejected below a *p* value of 0.05. Details of statistical tests are available in Table 1; superscript letters throughout the text indicate the corresponding statistic in the table.

**Table 1.** Paper statistics

	**Figure**	**Comparison**	**Data structure (Shapiro–Wilk normality test unless otherwise stated)**	**Type of test**	**Statistic**	**Confidence, 95% CI**
a	1*C*	NTG vs hAPP	Normal distribution (D’Agostino & Pearson normality test chosen due to multiple duplicate values)	Unpaired two-tailed *t* test	*t* = 0.04476 df = 29	*p* = 0.9646; CI: −0.8191 to 0.7841
b	1*D*	NTG vs hAPP	Normal distribution (D’Agostino & Pearson Normality Test chosen due to multiple duplicate values)	Unpaired two-tailed *t* test	*t* = 0.3928 df = 27	*p* = 0.6975; CI: −0.9669 to 1.425
c	1*E*	NTG vs hAPP	Normal distribution	Unpaired two-tailed *t* test	*t* = 3.093 df = 28	*p* = 0.0045; CI: −15.53 to -3.156
d	1*F*	NTG vs hAPP	Normal distribution	Unpaired two-tailed *t* test	*t* = 0.1181 df = 31	*p* = 0.9067; CI: −2.015 to 1.794
e1	2*B*	Ovarian status by hour interaction	Normal distribution	Mixed-model ANOVA	*F*_(22,88)_ = 2.05	*p* = 0.0097
e2	2*B*	Ovarian status effect	Normal distribution	Mixed Model ANOVA	*F*_(2,110)_ = 20.89	*p* < 0.0001
e3	2*B*	Metestrus/Diestrus vs Proestrus	Normal distribution	Bonferroni–Holm Corrected		*p* < 0.0001; CI: −18.615 to -9.899
e4	2*B*	Proestrus vs Gnx	Normal distribution	Bonferroni–Holm Corrected		*p* = 0.0156; CI: 4.538 to 32.026
e5	2*B*	Metestrus/Diestrus vs Gnx	Normal distribution	Bonferroni–Holm Corrected		*p* = 0.5581; CI: −9.719 to 17.769
f1	2*C*	Proestrus after vs before	Normal distribution	Paired one-tailed *t* test	*t* = 3.851 df = 2	*p* = 0.0307; CI: 8.136 to ∞
f2	2*C*	Before E2 surge	Normal distribution	One-way ANOVA	*F*_(2,8)_ = 0.2880	*p* = 0.7572
f3	2*C*	After E2 surge	Normal distribution	One-way ANOVA	*F*_(2,8)_ = 8.534	*p* = 0.0104
f4	2*C*	After surge: metestrus/diestrus vs proestrus	Normal distribution	Bonferroni–Holm Corrected	*t* = 3.129 df = 8	*p* = 0.0421; CI: −66.77 to -1.234
f5	2*C*	After surge: proestrus vs Gnx	Normal distribution	Bonferroni–Holm Corrected	*t* = 3.986 df = 8	*p* = 0.0121; CI: 9.427 to 68.04
f6	2*C*	After surge: metestrus/diestrus vs Gnx	Normal distribution	Bonferroni–Holm Corrected	*t* = 0.4871 df = 8	*p* > 0.9999; CI: −24.57 to 34.04
g1	3*A*	Ovarian status by genotype interaction	Normal distribution	Two-way ANOVA	*F*_(2,34)_ = 1.365	*p* = 0.2690
g2	3*A*	Ovarian status effect	Normal distribution	Two-way ANOVA	*F*_(2,34)_ = 1.776	*p* = 0.1846
g3	3*A*	Genotype effect	Normal distribution	Two-way ANOVA	*F*_(1,34)_ = 0.01396	*p* = 0.9066
g4	3*A*,*B*	Training vs testing latency	Normal distribution	Linear regression	*F*_(1,35)_ =1.559	*p* = 0.2202; *p* = 0.1049-0.7498 for each experimental group
h1	3*B*	Ovarian status by genotype interaction	Normal distribution	Two-way ANOVA	F_(2,31)_ = 4.88	*p* = 0.0144
h2	3*B*	Ovarian status effect	Normal distribution	Two-way ANOVA	*F*_(2,31)_ = 0.497	*p* = 0.631
h3	3*B*	Genotype effect	Normal distribution	Two-way ANOVA	*F*_(1,31)_ = 2.323	*p* = 0.1376
h4	3*B*	hAPP-High E/P vs hAPP-Low E/P	Normal distribution	Bonferroni–Holm Corrected	*t* = 3.126 df = 6	*p* = 0.041; CI: −186.5 to −22.73
h5	3*B*	hAPP-High E/P vs NTG-High E/P	Normal distribution	Bonferroni–Holm Corrected	*t* = 3.969 df = 6	*p* = 0.022; CI: −211.1 to −50.07
h6	3*B*	hAPP-Low E/P vs NTG-Low E/P	Normal distribution	Bonferroni–Holm Corrected	*t* = 1.149 df = 10	*p* = 0.275; CI: −34.47 to 107.8
h7	3*B*	Gnx hAPP vs Gnx NTG, Reference for Cycling Mice	Normal distribution	Unpaired two-tailed *t* test	*t* = 0.04699 df = 15	*p* = 0.9631; CI: −71.92 to 68.82
i1	3*C*	hAPP: % Time in High E/P & Latency	Normal distribution	Linear regression	*R* ^2^ = 0.7144	*p* = 0.0041; CI (slope): −5.078 to −1.411
i2	3*C*	NTG: % Time in High E/P & Latency	Normal distribution	Linear regression	*R* ^2^ = 1.334e-005	*p* = 0.9910; CI (slope): −3.702 to 3.664
j1	4*A*	NTG-High E/P: Novel vs Familiar	Normal distribution	Paired one-tailed *t* test	*t* = 3.367 df = 9	*p* = 0.0042; CI: 0.5634 to ∞
j2	4*A*	NTG-Low E/P: Novel vs Familiar	Normal distribution	Paired one-tailed *t* test	*t* = 7.912 df = 3	*p* = 0.0021; CI: 1.212 to ∞
j3	4*A*	hAPP-High E/P: Novel vs Familiar	Normal distribution	Paired one-tailed *t* test	*t* = 0.9654 df = 11	*p* = 0.1775; CI: −0.3776 to ∞
j4	4*A*	hAPP-Low E/P: Novel vs Familiar	Familiar: normal distribution; Novel : not-normal (*p* = 0.0167)	Paired one-tailed *t* test	*t* = 2.066 df = 6	*p* = 0.0422; CI: 0.05542 to ∞
j5	4*B*	hAPP-High E/P vs theoretical mean (31.33)	Normal distribution	Two-tailed one sample *t* test	*t* = 2.525 df = 11	*p* = 0.0282; CI: −6.665 to -0.4568
j6	4-1	Ovarian status effect	Normal distribution	Two-way ANOVA	*F*_(1,35)_ = 0.5209	*p* = 0.4752
j7	4-1	Genotype effect	Normal distribution	Two-way ANOVA	*F*_(1,35)_ = 1.448	*p* = 0.2370
j8	4-1	Ovarian status by genotype interaction	Normal distribution	Two-way ANOVA	*F*_(1,35)_ = 0.17	*p* = 0.6826
k1	5	Ovarian status by genotype interaction	Normal distribution except hAPP Met/Di (*p* = 0.0382)	Two-way ANOVA	*F*_(2,41)_ = 0.9277	*p* = 0.4036
k2	5	Ovarian status effect	Normal distribution except hAPP Met/Di (*p* = 0.0382)	Two-way ANOVA	*F*_(2,41)_ = 3.501	*p* = 0.0395
k3	5	Genotype effect	Normal distribution except hAPP Met/Di (*p* = 0.0382)	Two-way ANOVA	*F*_(1,41)_ = 36.95	*p* < 0.0001
k4	5	hAPP-High E/P vs hAPP-Low E/P	Normal distribution except hAPP Met/Di (*p* = 0.0382)	Unpaired two-tailed *t* test	*t* = 2.559 df = 8	*p* = 0.0337; CI: −1211 to −62.90
l	6*B*	hAPP Met/Di vs Proestrus	Normal distribution	Unpaired two-tailed *t* test	*t* = 4.319 df = 10	*p* = 0.0015; CI: −0.8811 to −0.2814
m	6*D*	hAPP Met/Di vs Proestrus	Normal distribution	Unpaired two-tailed *t* test	*t* = 1.107 df = 11	*p* = 0.2921; CI: −0.1201 to 0.3628

n	6*F*	hAPP Met/Di vs Proestrus	Normal distribution	Unpaired two-tailed *t* test	*t* = 1.798 df = 10	*p* = 0.1024; CI: −0.4675 to 0.04995
o	6*G*	hAPP Met/Di vs Proestrus	Normal distribution	Unpaired two-tailed *t* test	*t* = 0.4533 df = 11	*p* = 0.6591; CI: −0.3629 to 0.2389
p	6*H*	hAPP Met/Di vs Proestrus	Normal distribution	Unpaired two-tailed *t* test	*t* = 0.149 df = 11	*p* = 0.8842; CI: −0.5582 to 0.4874
q	7*A*	Genotype by Treatment Interaction	Normal distribution (D’Agostino & Pearson normality test chosen due to multiple duplicate values)	Linear mixed-model		*p* = 0.017; CI: −829.9 to -80.8
r1	7*B*	Genotype by treatment interaction	Normal distribution for NTG and hAPP Veh; *N* too small to determine if Gaussian for NTG and hAPP E2 (D’Agostino & Pearson normality test chosen due to multiple duplicate values)	Two-way ANOVA	*F*_(1,26)_ = 5.157	*p* = 0.0317
r2	7*B*	Genotype effect	Normal distribution for NTG and hAPP Veh; *N* too small to determine if Gaussian for NTG and hAPP E2 (D'Agostino & Pearson normality test chosen due to multiple duplicate values)	Two-way ANOVA	*F*_(1,26)_ = 7.982	*p* = 0.009
r3	7*B*	Treatment effect	Normal distribution for NTG and hAPP Veh; *N* too small to determine if Gaussian for NTG and hAPP E2 (D’Agostino & Pearson normality test chosen due to multiple duplicate values)	Two-way ANOVA	*F*_(1,26)_ = 0.7303	*p* = 0.4006
r4	7*B*	Gnx-E2: NTG vs hAPP	Normal distribution for NTG and hAPP Veh; *N* too small to determine if Gaussian for NTG and hAPP E2 (D’Agostino & Pearson normality test chosen due to multiple duplicate values)	Bonferroni–Holm Corrected	*t* = 3.701 df = 12	*p* = 0.006; CI: 301.7 to 1165
r5	7*B*	Gnx-Veh: NTG vs hAPP	Normal distribution for NTG and hAPP Veh; *N* too small to determine if Gaussian for NTG and hAPP E2 (D’Agostino & Pearson normality test chosen due to multiple duplicate values)	Bonferroni–Holm Corrected	*t* = 0.3877 df = 14	*p* = 0.7041; CI: −521.3 to 361.7
s1	7*C*	Gnx-E2: NTG vs hAPP	Categorical data	χ^2^ Test	Pearson χ^2^_(1,_*_n_*_= 14)_ = 7.7778	*p* = 0.005
s2	7*C*	Gnx-Veh: NTG vs hAPP	Categorical data	χ^2^ Test	Pearson χ^2^_(1,_*_n_*_= 16)_ = 0.2909	*p* = 0.590
t	7*D*	Gnx-hAPP: Veh vs E2	Normal distribution	Unpaired two-tailed *t* test	*t* = 2.239 df = 19	*p* = 0.0373; CI: −0.6 to −0.02019

## Results

### Ovarian cycles in female hAPP mice

Before examining effects of ovarian cycle stage on brain function, we verified that hAPP mice reliably enter all stages of the cycle. To this end, we investigated whether female hAPP mice exhibited regular reproductive cycles compared to NTG controls by vaginal cytology. Cycle stages were determined (Fig. 1*A*) and classified by their hormonal status (Fig. 1*B*). NTG and hAPP mice did not differ in their numbers of ovarian cycles over 3 weeks (Fig. 1*C*; *p* = 0.9646^a^) or in their average cycle length (Fig. 1*D*; *p* = 0.6975^b^).

Ovarian cycle stages result in specific changes in circulating gonadal hormones. To determine whether hAPP mice experience similar hormonal fluctuations compared with NTG mice, we classified each day of the cycle based on estradiol to progesterone ratios (Fig. 1-1) derived from previously established hormonal values (Walmer et al., 1992). Proestrus and estrus, the follicular-like stages (Allen, 1922), have a ratio of high estradiol and low progesterone (High E/P), whereas metestrus and diestrus, luteal-like stages, have a ratio of low estradiol and high progesterone (Low E/P; Fig. 1*B*). Interestingly, hAPP mice spent a greater percentage of time in High E/P (Fig. 1*E*,*G*; *p* = 0.0045^c^) despite similar cycle length compared to NTG mice. This alteration in cycle pattern did not affect overall fertility as NTG and hAPP females had litters of equivalent size (Fig. 1*F*; *p* = 0.9067^d^), though number of litters per mouse was not assessed. Thus, hAPP mice exhibited all phases of the reproductive cycle and normal fertility, but showed an increase in the follicular duration of the cycle, when estradiol levels are highest.

### Proestrus cycle stage (High E/P ratio) increases network hyper-synchrony in female hAPP mice

Estrogen-dominant stages increase network excitability and progesterone-dominant stages decrease it (Brinton et al., 2008; Spencer et al., 2008). Because hAPP mice (Verret et al., 2012; Martinez-Losa et al., 2018) and patients with AD (Amatniek et al., 2006; Vossel et al., 2013; Palop and Mucke, 2016) display brain network hyper-synchrony, we hypothesized that estrogen-dominant ovarian cycle stages, and specifically high estradiol levels, worsen network dysfunction and hyperexcitability in hAPP mice.

**Figure 2.**
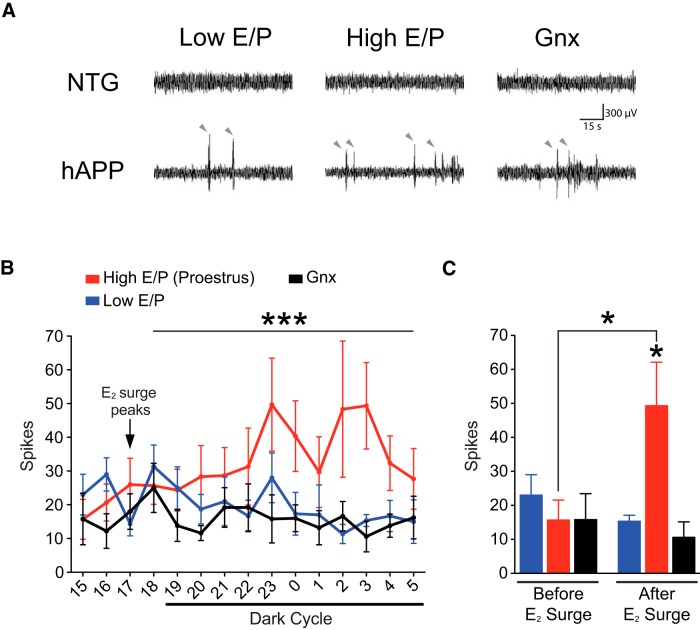
Proestrus increases spontaneous epileptiform activity in female hAPP mice. NTG and hAPP female mice were cycled to determine ovarian cycle stage (Pro, Est, Met, Di) and another group was Gnx. All groups underwent network activity analysis with EEG. ***A***, Representative EEG traces from NTG and hAPP female mice during Low E/P (metestrus/diestrus), High E/P (proestrus/estrus), and Gnx states. ***B***, Number of spikes graphed by hour from 15:00 to 05:00 in proestrus (red), Low E/P (metestrus/diestrus; blue), and Gnx (black) hAPP mice. Mixed-model ANOVA: ovarian status by hour interaction *p* < 0.01^e1^, ovarian status effect ***p < 0.0001^e2^; *p* < 0.0001^e3^ High E/P (proestrus) vs Low E/P (metestrus/diestrus), *p* < 0.05^e4^ High E/P (proestrus) versus Gnx. ***C***, Number of spikes during representative hours before (15:00) and after (03:00) E_2_ surge. One-way ANOVA: after E_2_ surge, *p* = 0.01^f3^, *p < 0.05 versus Low E/P (metestrus/diestrus)^f4^ and Gnx^f5^ or as indicated by bracket^f1^. (*n* = 3–5 mice per genotype, age 2.5–4 months). Data are mean ± SEM.

To test this hypothesis, we monitored neural network function in hAPP female mice by EEG. Female mice were classified as being in High E/P or Low E/P according to ovarian cycle stage; for the High E/P group, we further focused our analysis on mice in proestrus, the periovulatory period, which is accompanied by a rapid and characteristic surge in estradiol levels in early evening (Merry and Holehan, 1994). As expected, hAPP mice showed aberrant network hyper-synchrony (Fig. 2*A*). To determine whether epileptiform spikes specifically increase following the proestrus surge of estradiol, we quantified the number of spikes during the hours before and after the estradiol surge and compared hAPP mice in proestrus to hAPP mice in metestrus and diestrus. We found that epileptiform spikes significantly increased in the proestrus group in the hours following the early evening surge in estradiol (Fig. 2*B*; ovarian status by hour interaction *p* = 0.0097^e1^; ovarian status effect *p* < 0.0001^e2^; Fig. 2*C*; *p* = 0.0307^f1^). In contrast, aberrant network activity did not increase in metestrus/diestrus mice and was decreased compared to proestrus mice (Fig. 2*B*; *p* < 0.0001^e3^; Fig. 2*C*; *p* = 0.0421^f4^). Thus, an increase in endogenous estradiol following proestrus resulted in increased network excitability in female hAPP mice.

We compared intact hAPP mice to those with ovarian hormone depletion via gonadectomy (Gnx) to further dissect whether Low E/P stages may contribute to attenuating network dysfunction. Interestingly, the number of spikes did not differ between Low E/P and Gnx hAPP mice, either before or after the estradiol surge (Fig. 2*B*; *p* = 0.5581^e5^; Fig. 2*C*; *p* > 0.9999^f6^). These data, along with the specific increase in spikes following the estradiol surge, suggest that circulating gonadal hormones high in estrogen drive exacerbation of neural network dysfunction in hAPP mice.

### Ovarian cycle stage modulates fear memory impairments in female hAPP mice

**Figure 3.**
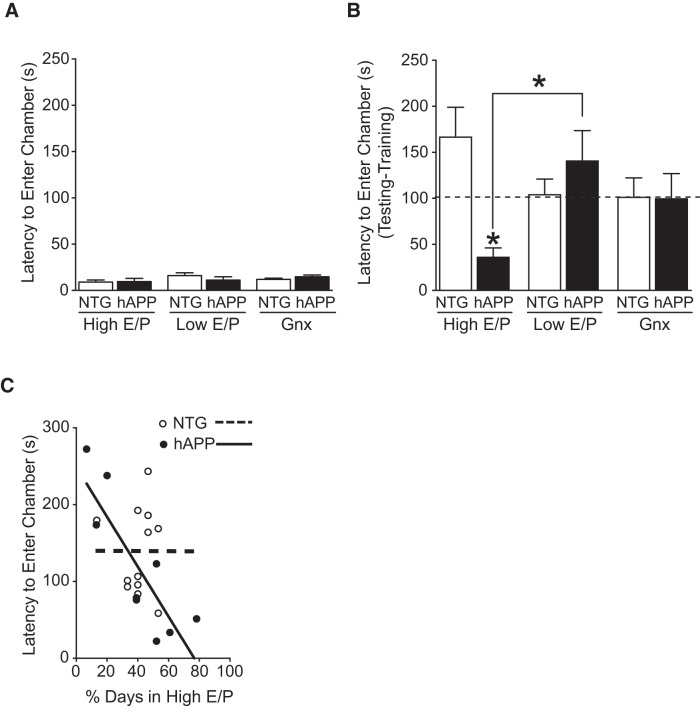
High E/P ovarian cycle stages, proestrus and estrus, worsen fear memory in female hAPP but not NTG mice. NTG and hAPP female mice were cycled to determine ovarian cycle stage (Pro, Est, Met, Di) and another group was Gnx. All groups were tested in the passive avoidance task. ***A***, Latency to enter the dark chamber during training in NTG and hAPP female mice by ovarian status: High E/P (proestrus/estrus), Low E/P (metestrus/diestrus), and Gnx. ***B***, Latency to enter the dark chamber during testing (minus latency during training) in NTG and hAPP female mice by ovarian status. Two-way ANOVA: ovarian status by genotype interaction, *p* < 0.05^h1^, *p < 0.05 versus NTG^h5^ or as indicated by bracket^h4^. Dashed line indicates average of Gnx group. ***C***, Correlation between percentage of time spent in High E/P (proestrus/estrus) over a 3 week period and latency to enter the dark chamber in intact mice; *R* ^2^ = 0.714. (*n* = 4–9 mice per group for ***A*** and ***B***; *n* = 9–12 mice per group for ***C***; age 2.5-4 months). Data are mean ± SEM.

Coordinated neural networks are key substrates of cognition (Mesulam, 1990; Sporns, 2013). Because aberrant network activity interferes with mechanisms of cognition (Palop and Mucke, 2016), and cognitive deficits are a primary clinical manifestation of AD, we investigated whether effects of ovarian cycle stage on network function extend to learning and memory. We tested cognition in NTG and hAPP mice using a passive avoidance task that measures contextual fear memory in mice, represented by the latency to re-enter the dark chamber during testing, where mice received an electric shock 24 h prior during training. There were no differences between groups in latency to cross during training and no significant correlations between latencies for training and testing (Fig. 3*A* ^g1-4^), indicating a lack of relationship between baseline activity on testing.

All groups of NTG and hAPP mice showed increased latency during testing, indicating learning. Notably, compared with Low E/P hAPP mice (metestrus/diestrus), High E/P hAPP mice (proestrus/estrus) showed profound memory deficits (Fig. 3*B*; ovarian status by genotype interaction *p* = 0.0144^h1^; High E/P versus Low E/P hAPP p = 0.041^h4^). Consistent with the network hyperexcitability results, High E/P dramatically worsened memory in hAPP mice. These data suggest that ovarian cycle stages high in estradiol cause or exacerbate cognitive dysfunction in female hAPP mice.

To further dissect whether High E/P stages worsen cognition in hAPP mice relative to NTG performance, we compared intact NTG and hAPP mice to those with gonadal hormone depletion via Gnx. Compared with High E/P NTG mice, High E/P hAPP mice had severe memory deficits (Fig. 3*B*; High E/P NTG versus High E/P hAPP, *p* = 0.022^h5^).

Remarkably, both Low E/P cycle stages (metestrus, diestrus) and Gnx abolished cognitive differences between NTG and hAPP female mice (Fig. 3*B*; Low E/P NTG vs Low E/P hAPP, *p* = 0.275^h6^; Gnx NTG vs Gnx hAPP, *p* = 0.963^h7^). Thus, deficits observed in female hAPP mice are in large part governed by reproductive cycle stage. Specifically, High E/P stages induce or worsen deficits in the female hAPP brain.

### Increased time spent in proestrus and estrus (High E/P ratio) correlates with worsened cognitive impairments in female hAPP mice

Because female hAPP mice spent more time in High E/P cycle stages (Fig. 1), we wondered whether increased time in High E/P might be associated with the degree of cognitive deficits in the passive avoidance task. To investigate this, we examined the relationship between latency to enter the dark chamber in the passive avoidance task and the percentage of days spent in High E/P cycle stages over a 3 week period, for each intact mouse. Spending a greater percentage of time in High E/P closely correlated with worse memory impairments in hAPP females (Fig. 3*C*; *p* = 0.0041^i1^, *R* ^2^=0.7144). In contrast, there was no such correlation in NTG mice (Fig. 3*C*; *p* = 0.9910^i2^). This suggests that more time in High E/P stages increases pathophysiology in hAPP female mice and could be detrimental to the AD brain.

### Proestrus and estrus (High E/P ratio) worsen spatial and working memory in female hAPP but not NTG mice

**Figure 4.**
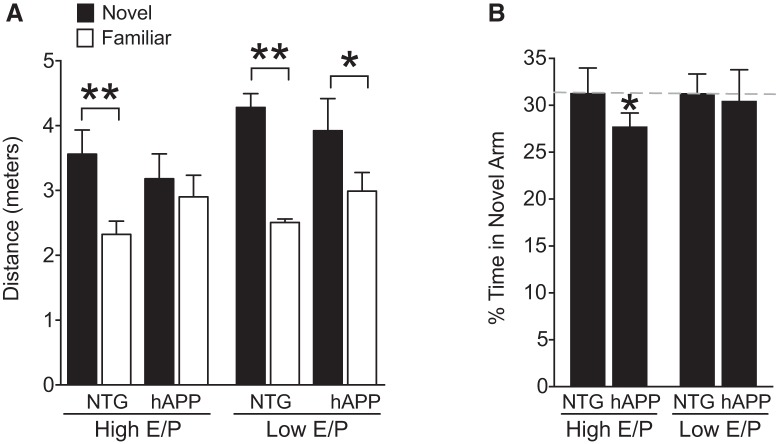
Proestrus and estrus, High E/P ovarian cycle stages, worsen spatial and working memory in female hAPP but not NTG mice. NTG and hAPP female mice were cycled to determine ovarian cycle stage (Pro, Est, Met, Di). All groups were then tested in the two-trial Y-maze that assesses exploration of a familiar and novel arm of the maze. ***A***, Distance (meters) traveled in novel (black bars) and familiar (white bars) arms of the two-trial Y-maze during testing. Total distance did not differ across groups (Fig. 4-1). ***B***, Percentage of time spent in the novel arm. **p < 0.01^j1-2^, *p < 0.05^j5^ as indicated by brackets (***A***) or versus NTG values (two-tailed one-sample *t* tests) in ***B***. Dashed gray line is NTG average of percentage time in novel arm (*n* = 5–13 mice per group, age 3.5–5.5 months). Data are mean ± SEM. See also Figure 4-1.

To determine whether High E/P cycle stages similarly worsen other forms of cognition, we tested NTG and hAPP female mice in the two trial Y-maze, a non-aversive task that tests working and spatial memory. As expected, NTG mice in all cycle stages traveled a greater distance in the novel arm than the familiar arm (Fig. 4*A*; Novel vs Familiar: High E/P NTG, *p* = 0.0042^j1^; Low E/P NTG, *p* = 0.0021^j2^) indicating increased exploration of the novel arm. Similar to NTG mice, Low E/P hAPP mice (metestrus and diestrus) also traveled a greater distance in the novel arm (Fig. 4*A*; Novel vs Familiar, *p* = 0.0422^j4^). In contrast, High E/P hAPP mice (proestrus and estrus) showed no difference in the distance traveled in the novel compared with the familiar arm and spent significantly less time in the novel arm compared with the other groups, indicating impaired memory (Fig. 4*A*; Novel vs Familiar, *p* = 0.1775^j3^; Fig 4*B*; *p* = 0.0282^j5^). There was no difference in total distance traveled in any of the groups, indicating differences seen in distance traveled reflect spatial and working memory and not increased movement or hyperactivity in any group (Fig. 4-1^j6-8^). The phenotype of hyperactivity of hAPP mice in the open field did not appear to translate into the closed environment of the passive avoidance apparatus or the two-trial Y-maze, because there was no evidence of increased baseline activity in the training phase of mice in either task. Thus, in tests that probe different domains of cognitive function across independent cohorts, the ovarian cycle stages of proestrus and estrus (High E/P) worsened cognition in female hAPP mice.

10.1523/ENEURO.0132-17.2018.f4-1 Figure 4-1 Total distance traveled in the two trial Y-maze does not differ between groups. Total distance (meters) traveled in the two trial Y-maze during testing of NTG and (hAPP transgenic mice grouped by those in high estrogen phase (High E/P) status (proestrus/estrus) and low estrogen phase (Low E/P; metestrus/diestrus). Two-way ANOVA: ovarian phase status effect, *p* = 0.475^j6^; genotype effect, *p* = 0.237^j7^; interaction effect, *p* = 0.683^j8^. *n* = 6–14 mice per group, age 3.5–5.5 months. Download Figure 4-1, EPS file

### Proestrus and estrus (High E/P) worsen hyperactivity in female hAPP mice

**Figure 5.**
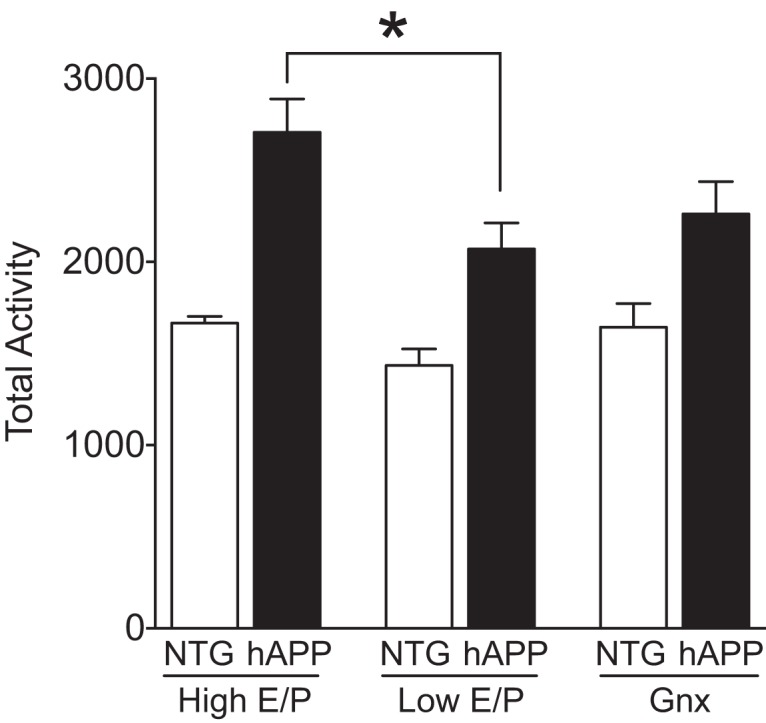
Proestrus and estrus, High E/P cycle stages, worsen locomotor hyperactivity in female hAPP but not NTG mice as detected in the context of an open field. NTG and hAPP female mice were cycled to determine ovarian cycle stage (Pro, Est, Met, Di) and another group was Gnx. All groups underwent testing in the open-field apparatus. Total number of movements during exploration of an open field in NTG and hAPP female mice by ovarian status: High E/P (proestrus/estrus), Low E/P (metestrus/diestrus), and Gnx. Two-way ANOVA: ovarian status effect, *p* < 0.05^k2^; genotype effect, *p* < 0.0001^k3^. *p < 0.05 as indicated by bracket^k4^. (*n* = 3–11 mice per group, age 2.5–4 months). Data are mean ± SEM.

We then tested whether High E/P cycle stages similarly exacerbate other behavioral abnormalities in hAPP mice using the open field test, which measures exploration and locomotor activity. As expected, total activity was increased in hAPP mice compared with NTG controls, regardless of cycle stage (Fig. 5; Genotype effect, *p* < 0.0001^k3^). In parallel with worsening of memory, High E/P (proestrus, estrus) increased hyperactivity in the open field compared with Low E/P (metestrus, diestrus) hAPP mice (Fig. 5; *p* = 0.0337^k4^). Thus, in addition to their effects on cognition, the ovarian High E/P cycle stages also worsened behavioral impairments in female hAPP mice when tested in the context of an open field.

### Ovarian cycle stage affects levels of pathogenic proteins in hAPP mice

Levels of Aβ are known to fluctuate with circadian rhythms (Bateman et al., 2007; Roh et al., 2012), but whether they fluctuate across the endogenous ovarian cycle has not been investigated. To assess whether the reproductive cycle alters levels of Aβ or other key proteins implicated in the pathogenesis of AD, we measured levels of Aβ_1-42_, hAPP, total Tau, and phosphorylated-Tau (P-Tau) in the hippocampus across ovarian cycle stages.

**Figure 6.**
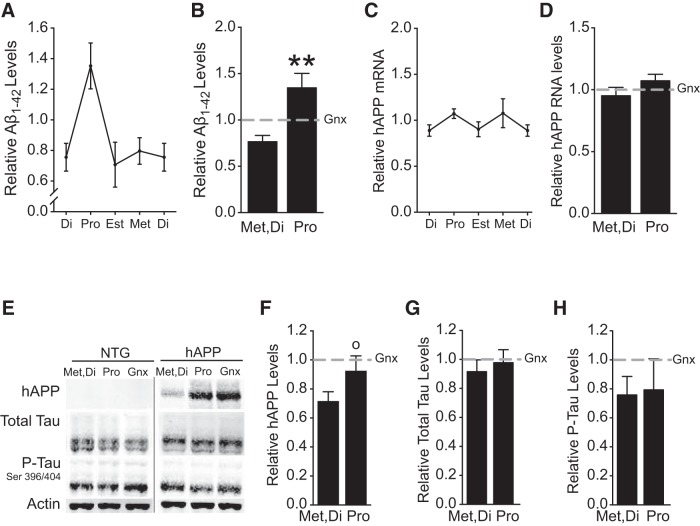
Levels of Aβ_1-42_ surge in the hippocampus of hAPP female mice during proestrus. NTG and hAPP female mice were cycled to determine ovarian cycle stage (Pro, Est, Met, Di) and another group was Gnx. Hippocampal homogenates were assessed for hAPP and pathogenic proteins related to AD. ***A***, Aβ_1-42_ levels determined by ELISA in hAPP mice at each stage of the ovarian cycle. ***B***, Aβ_1-42_ levels during metestrus/diestrus compared to proestrus (*n* = 4–13 mice per group, age 2.5–4 months). **p < 0.01 versus metestrus/diestrus.^l^ ***C***, Relative hAPP mRNA levels determined by qPCR in hAPP mice at each stage of the ovarian cycle. ***D***, Relative hAPP mRNA levels during metestrus/diestrus compared to proestrus (*n* = 4–14 mice per group, age 2.5–4 months). ***E***, Representative Western blot showing hippocampal levels of hAPP, total mouse tau, phosphorylated mouse tau, and the loading control actin, in metestrus/diestrus, proestrus, and Gnx NTG and hAPP mice. ***F–H***, Relative hAPP (***F***), total Tau (***G***), and phosphorylated Tau (***H***) protein levels determined by Western blot in hAPP mice during metestrus/diestrus compared to proestrus. All values are relative to Gnx hAPP levels as represented by dashed gray lines. (*n* = 7–13 mice per group for ***F***–***H***; age 2.5–4 months). Data are mean ± SEM.

Levels of Aβ_1-42_ sharply increased during proestrus relative to other cycle stages (Fig. 6 *A,B*; p = 0.0015^l^). However, neither mRNA levels (Fig. 6 *C,D*; *p* = 0.2921^m^) nor protein levels (Fig. 6 *E*,*F*; *p* = 0.1024^n^) of full-length hAPP changed. Because abnormal network excitation increases hAPP processing and Aβ production (Kamenetz et al., 2003; Cirrito et al., 2005) these findings suggest that the proestrus surge in network excitation mediates the transient increase in Aβ_1-42_. Levels of Tau (Fig. 6 *E,G*; *p* = 0.65910) and P-Tau (Fig. 6 *E*,*H*; *p* = 0.8842^p^) did not change during proestrus relative to metestrus and diestrus in female hAPP mice.

### Estradiol worsens seizure susceptibility and death in gonadectomized hAPP female mice but not in gonadectomized NTG mice

Because High E/P (proestrus) markedly worsened neural function in hAPP mice compared with Low E/P (metestrus and diestrus), and these Low E/P cycle stages paralleled the Gnx, hormone depleted state, we hypothesized that 17β-estradiol, the major biologically active, circulating gonadal estrogen during the reproductive cycle, increases network hyperexcitability in hAPP mice. To test this, we used a seizure model of overactive network activity. We treated Gnx mice with vehicle (Veh) or a single dose of 17β-estradiol benzoate (E_2_; 5 μg, i.p.) that results in proestrus range levels (Akinci and Johnston, 1997); 24 h afterward we measured seizure susceptibility to PTZ (35 mg/kg, i.p.), which induces network hyperexcitability and hyper-synchrony via blockade of GABA-A receptors. Latency to reach each stage of seizure activity, which ranged from a score of 1 (pause in movement) to 8 (death), was measured as described previously (Roberson et al., 2007).

**Figure 7.**
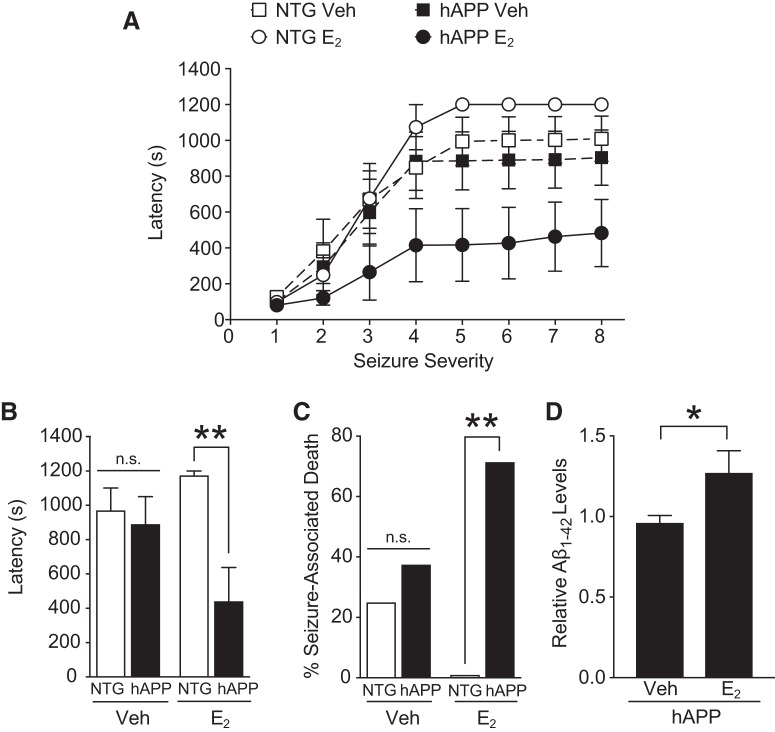
Estradiol treatment worsens excitotoxicity and seizure-associated death in gonadectomized female hAPP mice and attenuates these measures in gonadectomized female NTG mice. Gnx NTG and Gnx hAPP female mice were treated (i.p.) with 100 μl vehicle (Veh) or 5 μg in 100 μl of 17β-estradiol benzoate (E_2_), a dose that mimics *in vivo* levels of estradiol during proestrus (Akinci and Johnston, 1997); 24 h later, mice were injected with 35 mg/kg (i.p.) of GABA-A receptor blocker PTZ. Behavioral activity using the Racine’s seizure scale was monitored for 20 min and scored from 1 (pausing) to 8 (death). ***A***, Latency to reach increasing levels of seizure severity following PTZ injection in Gnx NTG and Gnx hAPP female mice treated with Vehicle or E_2_. Increased latencies indicate greater resistance to seizure, whereas decreased latencies indicate greater susceptibility to seizure. Mixed model ANOVA: genotype by treatment effect, *p* < 0.05^q^. ***B***, Latency to reach increasing levels of seizure severity in Gnx NTG and Gnx hAPP female mice treated with Vehicle or E_2_, averaged across late seizure stages (Stages 4–8). Two-way ANOVA: genotype by treatment interaction, *p* < 0.05^r1^, genotype effect, *p* < 0.01^r2^. **p < 0.01 as indicated by bracket.^r4^ ***C***, Percentage seizure-induced death in NTG and hAPP female mice treated with Vehicle or E_2_. **p < 0.01^s1^. ***D***, Aβ_1-42_ levels of hAPP mice treated with either Vehicle or E_2_ 24h before PTZ-induced seizures. *p < 0.05^t^ (*n* = 7–8 mice per group for ***A***–***C***, age 6–7 months; *n* = 10–11 mice per group for ***D***, age 2–4.5 months). Data are mean ± SEM.

E_2_ treatment augmented seizure severity in Gnx hAPP compared to Gnx NTG mice (Fig. 7*A*; genotype by treatment interaction, *p* = 0.017^q^). This difference was particularly evident in later stages of seizure activity (Stages 4–8; Fig. 7*B*; *p* = 0.006^r4^), when network dysfunction is more severe and includes tonic or clonic motor dysfunction.

Vehicle-treated Gnx NTG and Gnx hAPP mice did not differ in seizure susceptibility (Fig. 7 *A,B*; *p* = 0.7041^r5^). Consistent with effects of Gnx on cognition and network dysfunction, these data suggest that depleting ovarian hormones decreases vulnerability to hyperexcitability in female hAPP mice, and that the observed deficits in intact, female hAPP mice may be driven by effects of gonadal hormones, specifically estradiol.

E_2_ treatment in Gnx NTG mice nearly eliminated death because of PTZ-induced seizures. Conversely, E_2_ treatment in Gnx hAPP mice caused mortality in the majority of mice (70%) following PTZ injection (Fig. 7*C*; *p* = 0.005^s1^). In contrast to E_2_, vehicle treatment in Gnx mice reduced differences in seizure-associated death between NTG and hAPP mice (Fig. 7*C*; *p* = 0.590^s2^). Thus, estradiol treatment aggravated network excitability in Gnx hAPP, but not Gnx NTG female mice.

### Estradiol treatment in gonadectomized hAPP female mice increases Aβ1-42 levels following PTZ-induced seizures

To further explore estradiol-mediated aggravation of network dysfunction, we tested whether the hormone increases Aβ_1-42_ following seizures. E_2_ treatment increased Aβ_1-42_ in Gnx hAPP mice following the induction of seizures (Fig. 7*D*; *p* = 0.0373^t^). These data suggest that estradiol, increased network dysfunction itself, or the combination can acutely increase Aβ_1-42_.

## Discussion

This study demonstrates that ovarian functions modulate a range of AD-related deficits in female hAPP mice. Estrogen-dominant cycle stages (High E/P ratio; proestrus and estrus) worsened network dysfunction, learning and memory, and behavioral deficits in female hAPP mice, whereas progesterone-dominant stages (Low E/P ratio; metestrus and diestrus) and gonadectomy attenuated these measures. Detrimental effects of High E/P cycle stages were specific to hAPP mice and did not extend to NTG littermates. Based on our biochemical, pharmacological and EEG data, we propose estrogen-mediated increase in network excitability as a mechanism triggering a surge in activity-dependent Aβ_1-42_ production and worsened cognitive and network functions. All together, these studies spanning multiple independent cohorts show that ovarian-derived fluctuations in endogenous gonadal hormones critically modify cognition, network function, and pathogenic protein levels. Further, these data highlight the importance of considering female biology and hormonal effects when investigating models of human disease.

### hAPP/Aβ alters the pattern of ovarian cycles

Despite normal fertility, cycle progression, and duration, female hAPP mice spent increased time in proestrus and estrus, follicular-like stages of the ovarian cycle when the E/P ratio is high. Interestingly, the cycle alteration closely resembles that of mice with mutations in core circadian clock genes (Miller et al., 2004; Chu et al., 2013), and with lesions to regions of the hypothalamus involved in circadian timing (Wiegand and Terasawa, 1982). Circadian rhythm disturbances (Sterniczuk et al., 2010; Duncan et al., 2012) and other hypothalamic abnormalities, have been reported in a number of AD mouse models. Whether altered ovarian cycles are mediated by AD-related changes along the hypothalamic-pituitary-gonadal axis, and whether they are similarly disrupted in young women at risk for AD, remains to be determined.

Individual female mice with increased time in High E/P showed more severe memory impairments. This suggests that certain patterns of ovarian cycling, even with normal fertility, could contribute to the pathogenesis of disease. In support of this possibility, High E/P stages acutely worsened abnormal network function, and dysregulated network function contributes to cognitive deficits and AD progression. Interestingly, there was considerable interindividual difference in the percentage of time spent in High E/P stages, suggesting it contributes to the wide phenotypic variation observed in intact, female hAPP mice.

### Estrogen-dominant cycle stages worsen network dysfunction in female hAPP mice

Abnormal network activity spiked in female hAPP mice following the estradiol surge on proestrus (High E/P), supporting a role for estradiol in increasing network excitability. Spikes increased several hours after the surge, suggesting that hormone–induced changes that involve genomic rather than rapid, membrane signaling may underlie these effects. Indeed, estradiol can increase excitability through a myriad of functions including increasing spine and synapse density (Woolley and McEwen, 1993; Yankova et al., 2001), augmenting NMDA receptor-mediated excitatory synaptic input (Woolley et al., 1997; Rudick and Woolley, 2001), and diminishing GABAergic inhibition (Murphy et al., 1998; Rudick and Woolley, 2001). These actions could exacerbate dysfunction in hAPP mice whose baseline hyper-synchrony and hyperexcitability render them vulnerable to detrimental effects of further excitation, including worsened cognition. Thus, the very mechanisms that increase excitability could enhance cognition in NTG mice but further impair hAPP mice because of their baseline network dysfunction and impaired synaptic homeostasis (Palop and Mucke, 2010), which would not allow appropriate compensation for gonadal hormone-induced excitation.

### Estrogen-dominant cycle stages worsen cognitive and behavioral deficits in female hAPP mice

High E/P stages (proestrus and estrus) increased cognitive deficits of female hAPP mice in both the aversive passive avoidance task involving the hippocampus and amygdala (Ambrogi Lorenzini et al., 1999), and the non-aversive two-trial Y-maze, (Dellu et al., 1992) involving the hippocampus and cortex. Notably, these brain regions, along with spatial learning and fear memory deficits, have long been observed to show dysfunction in AD (Vlcek and Laczo, 2014). Estradiol-mediated increases in network excitability may have contributed to these deleterious effects on multiple brain regions in female hAPP mice.

Remarkably, depletion of gonadal hormones via gonadectomy rescued AD-related deficits, and eliminated increased seizure susceptibility in female hAPP mice. This suggests that in hAPP-J20 female mice, deficits are largely dependent on ovarian-derived gonadal hormones, and specifically on the High E/P state of proestrus and estrus cycle stages.

The absence of a change in full-length hAPP mRNA across the ovarian cycle indicates that the observed alterations in pathogenic proteins, cognition, and network dysfunction are not mediated through effects on the hAPP transgene promoter. Because gonadal hormones modulate gene transcription through receptor-dependent and -independent mechanisms, it is important to ensure they do not exogenously activate the transgene promoter when examining female mice in transgenic mouse models of neurodegenerative disease.

### Levels of pathogenic Aβ1-42 surge following increased ovarian cycle-induced network excitability in hAPP mice

Levels of the pathogenic protein Aβ_1-42_ surged in female hAPP mice following increased abnormal network activity on proestrus. Several lines of evidence support a role for activity-dependent APP processing and Aβ secretion. In mouse models of AD, increasing synaptic (Kamenetz et al., 2003) and abnormal network (Cirrito et al., 2005) activities stimulate formation and secretion of Aβ, effects linked to NMDA receptor activation (Lesne et al., 2005). Given that proestrus (Galvin and Ninan, 2014) and particularly estradiol (Adams et al., 2004; Smith and McMahon, 2006; Snyder et al., 2011), stimulates synaptic localization and function of GluN2B-containing NMDA receptors, we propose that this estrogen-dominant cycle stage increases Aβ production by stimulating synaptic and network excitability in hAPP mice. Our data do not exclude potential contributions of estrogenic mechanisms that modulate γ-secretase (Jung et al., 2013) and α-secretase (Jaffe et al., 1994; Xu et al., 1998) observed in cell lines or primary cultures; importantly, we offer transient, estradiol-driven excitation of synaptic and network level functions as dominant mechanisms that increase Aβ production *in vivo*. Our studies were focused on young reproductively intact mice before the onset of plaque deposition (Mucke et al., 2000). Thus, whether the endogenous ovarian cycle stages alter plaque formation remains to be determined.

Effects of ovarian cycle stage compared with gonadectomy in mice that model AD or other dementias have not been investigated to our knowledge. In contrast to the focus of the current study, many have compared gonadectomy to chronic estradiol treatment (Levin-Allerhand et al., 2002; Zheng et al., 2002; Carroll et al., 2007, 2010; Dubal et al., 2012), administered to model hormone replacement therapy. Important distinctions between our study and the aforementioned are that our work focuses directly on previously unknown biology including (1) actions of endogenous ovarian cycle stages, (2) effects of ovarian cycle stage on network function and cognition, and (3) the impact of a subacute, estradiol pulse, that models the proestrus surge, on substrates of cognition in a mouse model of AD.

### A transient pulse of estradiol exacerbates excitotoxin-induced, abnormal network activity and increases Aβ1-42 levels in hAPP mice

Similar to the effects of proestrus on exacerbating abnormal network activity in hAPP female mice, a transient and subacute pulse of exogenous estradiol increased Aβ_1-42_ levels and excitotoxin-induced seizure susceptibility and severity. These findings support the hypothesis that estradiol acutely worsens the dysregulated neural network of the hAPP mouse brain. Of note, estradiol initially increased excitability in both NTG and hAPP mice, in line with its known excitatory functions, but attenuated seizure severity and death in NTG mice. Differing actions of estradiol in the NTG and hAPP brain suggest that it engages mechanisms that exert opposing effects in a normal versus dysregulated neural network. With this in mind, it is interesting to speculate that estrogen replacement in the Women’s Health Initiative Memory Study clinical trial (Shumaker et al., 2003, 2004; Espeland et al., 2004) may have increased clinical symptoms of dementia in a discrete subpopulation of women already undergoing the silent, “dysregulated network” phase that preceeds AD, but may have benefitted those undergoing normal aging with “normal networks”; this possibility remains to be tested. Our data provocatively suggest that estrogenic effects may aggravate the brain at risk for AD, but benefit a normal brain. This possibility could inform future clinical studies to determine whether estrogen replacement could harm or benefit the brain in women, depending on their individual AD risk.

Other hormones vary across the ovarian cycle. Interestingly, luteinizing hormone and follicle-stimulating hormone peak during proestrus and increase following Gnx (Kovacic and Parlow, 1972); however, because female hAPP mice with both reduced and elevated levels of these hormones showed equivalent cognitive and network functions, they are unlikely to govern substrates measured. Relatedly, progesterone peaks during metestrus and diestrus (Low E/P stages), cycle stages that show equivalent cognitive and network patterns to the Gnx, hormone-depleted state; this broadly suggests that increased excitation during High E/P stages probably results from estradiol-related gain of excitation rather than progesterone-related inhibition (Maguire et al.,2005; Wu et al., 2013). It is also worth noting that neuroactive derivatives of steroid hormones, neurosteroids (Frye, 2009; Panzica et al., 2012), may fluctuate across the reproductive cycle and play a role in estrogenic and other hormone-mediated effects.

### Studying the ovarian cycle may be relevant to human disease

Animal studies across numerous fields, and particularly in neuroscience (Zucker and Beery, 2010) disproportionately study males, in part because of the variable phenotypes in females probably related to their ovarian cycles. An emerging emphasis on increased representation of females in preclinical studies by the Institute of Medicine (Pankevich et al., 2011) and the National Institutes of Health (Clayton and Collins, 2014) raises new awareness and considerations (McCarthy et al., 2012). Should the ovarian cycle be incorporated into studies of females? Should gonads of males and females be removed to attenuate confounds of differential and fluctuating hormone action in the brain when including both sexes? The answers to these critical questions will vary according to the experimental questions, models investigated, and relevance of studies to the reproductive life stage of women.

Our study provides a fundamental model for performing rigorous research in adult female mice by investigating effects of the ovarian cycle in a model of AD. Considering that the pathogenesis of the disease begins decades before clinical diagnosis, women are likely to be undergoing reproductive cycling during the preclinical stages of the disease. Thus, investigating and understanding network activity, cognition, and other manifestations of brain function across the reproductive cycle in young women could reveal differential patterns in the brain at risk for AD compared with normal aging, and potentially open the door for preventative therapies and early treatment in women at risk for AD, a possibility that requires investigation in humans.

## Synthesis

Reviewing Editor: Margaret McCarthy, Univ of Maryland School of Medicine Decisions are customarily a result of the Reviewing Editor and the peer reviewers coming together and discussing their recommendations until a consensus is reached. When revisions are invited, a fact-based synthesis statement explaining their decision and outlining what is needed to prepare a revision will be listed below. The following reviewer(s) agreed to reveal their identity: Peng Lei.

Both reviewers continue to feel that the manuscript addresses an important topic and makes a useful contribution to the literature. However, they both expressed some additional concerns. I have reviewed these carefully and while I agree they are legitimate comments, I do not feel that additional experiments are required. Instead, I ask that you consider each comment carefully and make changes to the manuscript as appropriate or explain why not if you feel so. I am providing you the comments in the raw as I think that will give you the best sense of the reviewers intent. I realize this manuscript has already undergone multiple revisions and that you may feel frustrated with the protracted process but in the end the manuscript will be better for it. After receiving your response, I will make a final decision.

Reviewer Comments:

1) 24 h is NOT long-term memory - i don't have an issue with the tests specifically used but i do think it behoves the authors to acknowledge that they are investigating shorter-term memory and not long-term memory. Please see the work of Sheena Josselyn/Paul Frankland who also use contextual fear conditioning but with delays of up to 42 d or using more complicated cognitive testing (Bussy-Saksida touch screens, radial arm maze) that require many days of testing that can better assess true long-term memory. Given mice can live up to 2 y, it stands to reason that 24 h is not considered long-term memory.

2) I must be missing something as the e cycles are not different but yet the tg mice spent more time in proestrus? they must spend shorter time in another phase or have longer cycles.

3) Also the acronyms E/P(high) for E high and P low is confusing - please consider E(high)/P(low)

4) latency and hyperactivity: it's not really convincing that there is no difference in baseline "activity" which is measured by step down. this needs to be a true activity score within the apparatus itself - distance travelled for example. If the same animals performed all the beh tests then they can use OFT as a covariate in the passive avoidance.

5) it's not clear whether mice were tested in more than one task and/or when brains were collected after testing.

6) In Fig 5, hAPP mice showed hyperactivity in general, such differences were not presented in Fig4a, what would be the explanation? Also in Fig 5, it was stated that the n number is between 3-11 (page 39, line 977). Therefore one of these groups only has 3 mice which may not be statistically meaningful.

7) In human studies, the risk for women after menopause (reduced estrogen levels) to get AD is higher than men, and estrogen has been shown to be protective. However in current study, estrogen is implied to danger the cognitive function of mice. How to reconcile these findings?

8) Similarly, the age of mice after menopause is about 10 months old. If the authors are correct about the effect of ovarian cycle stages on cognition, they may need to test mice at that age?

9) Did the authors tested male mice at the same age to compare the cognitive status?

10) In Fig 6, did the authors perform WBs for Abeta1-42?
